# Luminescent Schiff-Base Lanthanide Single-Molecule Magnets: The Association Between Optical and Magnetic Properties

**DOI:** 10.3389/fchem.2019.00063

**Published:** 2019-02-06

**Authors:** Jérôme Long

**Affiliations:** Institut Charles Gerhardt, Equipe Ingénierie Moléculaire et Nano-Objets, Université de Montpellier, ENSCM, CNRS, Montpellier, France

**Keywords:** lanthanides, single-molecule magnet, multifunctional molecular materials, luminescence, anisotropy, crystal-field splitting

## Abstract

Luminescent Single-Molecule Magnets (SMM) belong to a new class of multifunctional molecule-materials that associate luminescence and slow relaxation of their magnetization within a single crystalline phase. We present in this mini-review the major advances that have been achieved in this new field over the last few years. More particularly, we will focus on the use of Schiff-base complexes in order to correlate magnetism and luminescence, as well as discussing the future outlooks of the field.

## Introduction

Nowadays, developing optimized molecule-based materials for future applications such as biomedicine (Horcajada et al., 2012; Long et al., 2016a), gas separation (Dechambenoit and Long, 2011), catalysis (Li et al., 2016), and quantum computing (Bogani and Wernsdorfer, 2008) frequently requires the association of different properties within a single-crystalline structure. Molecule-based materials benefit from specific assets related to their molecular nature, with respect to usual solid-state materials such as an unlimited structural diversity, weak density, optical transparency and the possibility to finely adjust and control their properties. Remarkably, coordination chemists have been at the front lines of science since the turn of the century, taking advantage of the respective intrinsic properties of metal ions and organic/inorganic ligands, to design original architectures with targeted functionalities. From a fundamental point of view, these unique molecular materials may display these properties independently, but the design of systems in which these functionalities strongly interact, constitutes the central objective for one property to control another.

Since the pioneering work of (Ishikawa et al., 2003), lanthanide-based Single-Molecule Magnets (SMM) have been investigated thoroughly because of their tremendous technological potential in high-density storage and quantum computing (Leuenberger and Loss, 2001; Woodruff et al., 2013; Liddle and van Slageren, 2015; Tang and Zhang, 2015; Ungur and Chibotaru, 2016). In such coordination complexes, an anisotropic barrier, Δ, originating from the interplay between the magnetic anisotropy and crystal-field splitting, opposes the reversal of the magnetization and leads to superparamagnetic-like behavior, comparable to that observed in magnetic nanoparticles. This feature may eventually give rise to a magnetic bistability that is strictly intrinsic to the molecular entity. Obviously, utilizing lanthanide ions constitutes a straightforward approach to implement simultaneous magnetic and luminescent properties because of their strong magnetic anisotropy and exceptional luminescence properties, dominated by *f*-*f* electronic transitions, which results in long-lived emission, narrow bandwidth, important Stokes shifts and high quantum yields (Bunzli and Piguet, 2005). While the collection of lanthanide SMM is growing exponentially, only a small percentage of those systems exhibit lanthanide luminescence and can therefore be viewed as multifunctional. In this mini-review, we discuss the use of Schiff-base ligands and associated complexes for the design of luminescent SMM, as well as providing future outlooks and directions in the field.

## Criteria to Design Lanthanide Luminescent SMM

Slow relaxation of the magnetization and lanthanide luminescence, arises in both cases from the subtle association between a defined lanthanide ion and appropriate ligand(s). Therefore, the SMM behavior depends on the nature of the lanthanide ion, such as its angular momentum value, *J*, its Kramers/non-Kramers character as well as the angular dependence of the 4*f* electronic density which can be oblate (flattened spheroid) or prolate (elongated spheroid) (Rinehart and Long, 2011; Ungur and Chibotaru, 2016). On the other hand, and considering simple electrostatic considerations, the crystal-field generated by the surrounding ligands could result in the formation of *m*_J_ states largely separated in energy. Since it is mostly the single-ion anisotropy that dominates the slow relaxation, Δ is therefore directly related to crystal-field splitting, through relaxation involving the first or higher excited states. Other mechanisms involved in spin-phonon coupling (Raman and direct processes) or Quantum tunneling of Magnetization (QTM) complicate this scenario however, by creating underbarrier relaxation paths. In this sense, significant advances have been achieved in recent years, with either coordination (Chen et al., 2016; Liu et al., 2016; Meng et al., 2018) or organometallic complexes (Chen et al., 2016; Ding et al., 2016; Gregson et al., 2016; Gupta et al., 2016; Goodwin et al., 2017; Guo et al., 2017) showing for instance magnetic hysteresis higher than liquid nitrogen boiling's temperature (Guo et al., 2018). Remarkably, while the QTM affects the magnetic relaxation at a low temperature, recent studies have highlighted the decisive role of molecular vibrations (spin-phonon coupling) at a higher temperature (Goodwin et al., 2017; Escalera-Moreno et al., 2018).

With regards to lanthanide luminescence, the parity and spin forbidden character of the *f*-*f* transitions usually require an indirect excitation through the use of sensitizer ligands that transfers the absorbed energy to the excited state of the lanthanide ion. Consequently, the ligand is of utmost importance since it directly dictates the coordination environment suitable for the slow relaxation of the magnetization, while ensuring an efficient luminescence sensitizing toward a specific lanthanide ion. Among the lanthanide series, Dy^3+^ ion represents one of the most promising candidates to design luminescent SMM, because of its large *J* = 15/2 value, its Kramers character leading to a doubly degenerated ground state and its oblate electronic density which could be easily stabilized by usual coordination chemistry ligands. Dy^3+^ luminescence could be observed both in the visible and Near-Infra Red (NIR) (Long et al., 2018b). To a lesser extent, the NIR emissive Yb^3+^ has also been widely employed to design luminescent SMM (Pointillart et al., 2015), but optimizing the slow relaxation remains more difficult to realize with respect to Dy^3+^. Tb^3+^ and Er^3+^ could also be employed to design luminescent SMM. Nevertheless, in practice they exhibit some drawbacks associated either to the Tb^3+^ non-Kramers nature (Ehama et al., 2013; Yamashita et al., 2013) or to the difficulty to observe Er^3+^-based luminescence (Ren et al., 2014). Historically, the first example of SMM simultaneously exhibiting a slow relaxation of the magnetization and a weak lanthanide luminescence was reported by Bi et al. in a tetranuclear calixarene dysprosium complex in 2009 (Bi et al., 2009). Following this, various ligand families (beta-diketonates, carboxylates, aromatic amines) have been successfully explored (Long et al., 2018b; Jia et al., 2019). We will however, next focus on another class belonging to Schiff bases and describe examples that go further than the simple observation of both properties.

## Luminescent Schiff-base SMM: Magneto-luminescence Correlation

**Figure 1:**
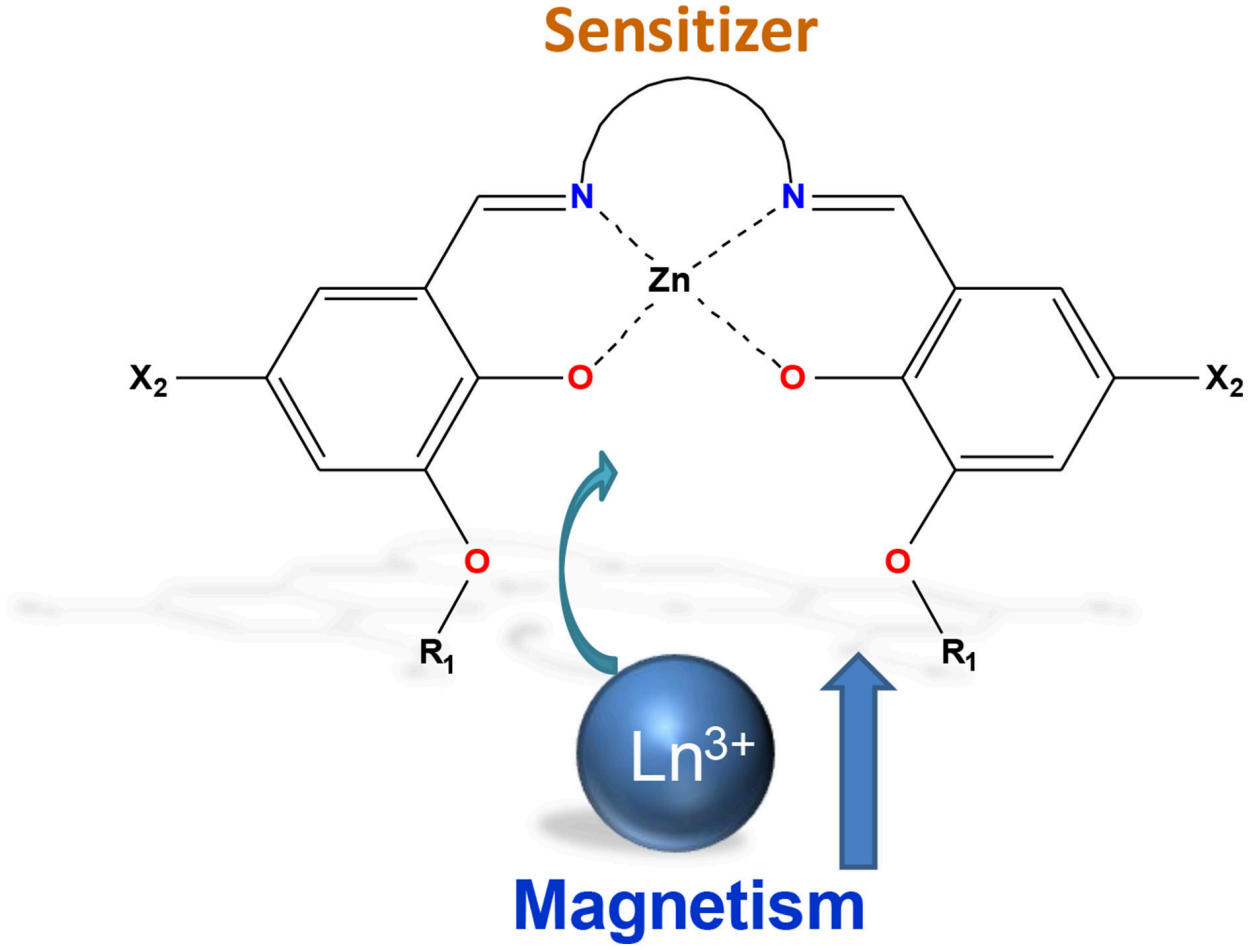
General scheme showing the combination of compartmental Schiff-base complex and Ln^3+^ ions.

**Figure 2:**
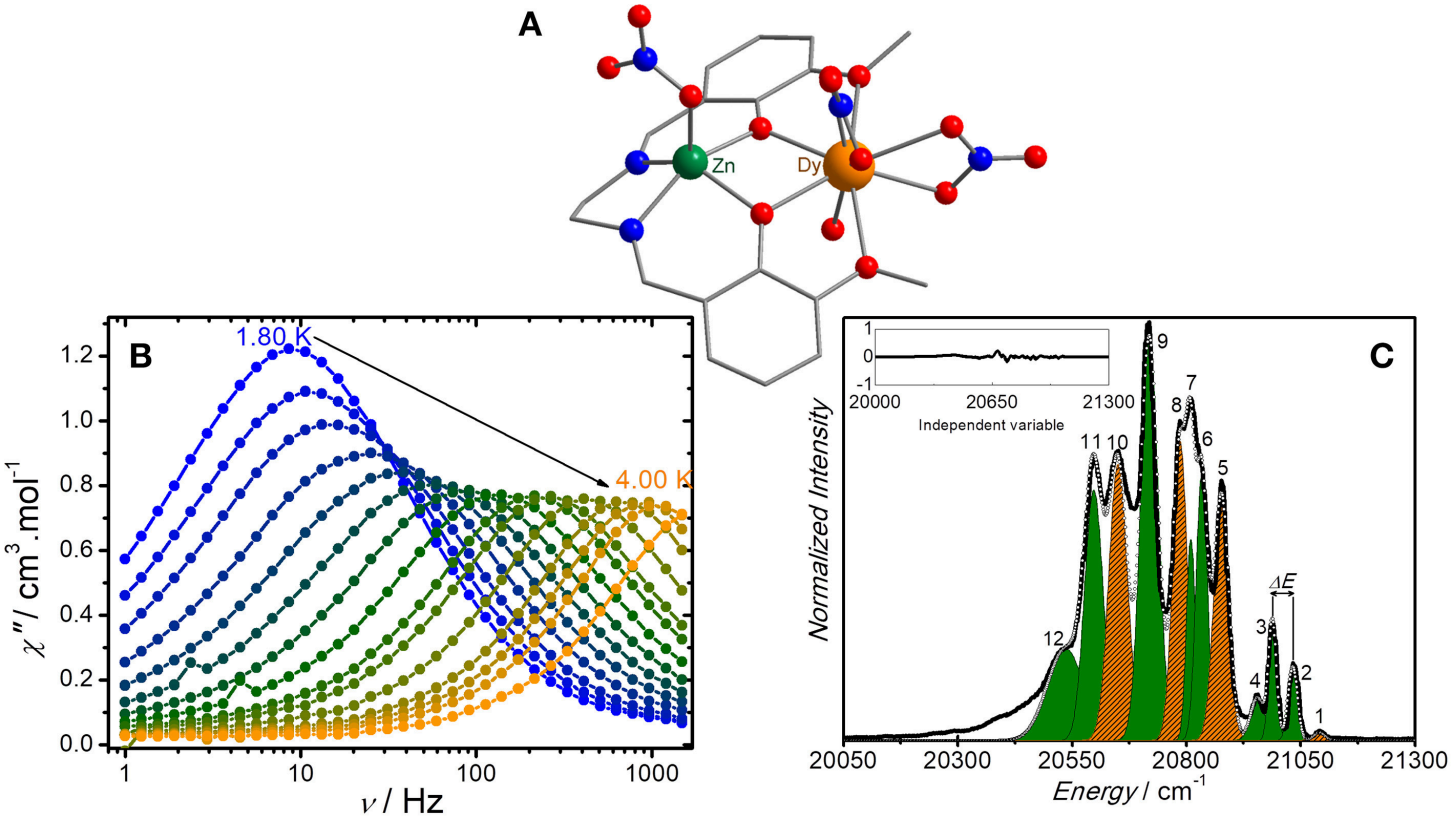
**(A)** Crystal structure of the dinuclear [Zn(NO_3_)L^1^Dy(NO_3_)_2_(H_2_O)] complex. **(B)** Frequency dependence of the out-of-phase susceptibility (χ”) obtained under a 900 dc field. **(C)** Magnification of the ^4^F_9/2_→^6^H_15/2_ emission transitions in the 20,050–21,300 cm^−1^ region (at 14 K) Multi-Gaussian function fit components arising from the first ^4^F_9/2_ Stark sublevel to the ^6^H_15/2_ multiplet in the energy interval. The fits regular residual plots (*R*^2^> 0.98) are shown in inset (Long et al., 2012, 2016b).

Schiff-base ligands are known as simple efficient sensitizers of Ln^3+^ (Yang et al., 2014; Andruh, 2015) while benefiting from a large tunability including the denticity, rigidity/flexibility, and selective coordination sites (Figure 1). Several 4*f* or 3*d*/4*f* luminescent complexes based on a myriad of Schiff-base ligands and incorporating various lanthanide ions such as Eu^3+^, Nd^3+^, Tb^3+^, Yb^3+^, have been reported since the beginning of the century (Wong et al., 2002, 2006; Yang and Jones, 2005; Burrow et al., 2009; Wang et al., 2009). Nevertheless, the occurrence of slow relaxation of magnetization in these complexes was either not achieved, due to the lack of magnetic anisotropy, or was not investigated. Thus, the first example of a bifunctional Schiff-base SMM was reported by our group in 2012 in a [Zn(NO_3_)L^1^Dy(NO_3_)_2_(H_2_O)] complex [H_2_L^1^: N,N'-bis(3-methoxysalicylidene)-1,2-diaminoethane)] and based on a simple compartmentalized ligand obtained from the condensation of *o*-vanillin and ethylenediamine (Long et al., 2012). The complex could be described as a dinuclear entity in which the connections between Zn^2+^ and Dy^3+^ ions are provided by phenolate bridges (Figure 2). Introduction of the diamagnetic Zn^2+^ ion clearly has two benefits: it increases the negative charge (basicity) of the phenolate moieties and in turn the crystal-field splitting of the lanthanide ion (Upadhyay et al., 2014) while it does not quench the rare-earth emission in the visible spectral window. At room temperature, both a broad emission band, ascribed to the zinc complex and the typical Dy^3+^ emission lines were observed, indicating a partial energy transfer toward the lanthanide ion. Lowering the temperature (14 K) results in the exclusive observation of well-resolved Dy^3+^ emission bands. Such feature is of prime interest since the emission lines involving the electronic transitions also involve the magnetic ground state ^6^H_15/2_ and as a consequence directly reflects its crystal-field splitting. Remarkably, such an approach was previously utilized in the 60's, using absorption spectroscopy for ytterbium (Buchanan et al., 1967) or dysprosium garnets (Grünberg et al., 1969) and this methodology was later extended by Cucinotta et al. (2012) to correlate luminescence and magnetic properties in the archetypical SMM Na[Dy(DOTA)(H_2_O)]·4H_2_O complex (Cucinotta et al., 2012). The emission spectrum for the complex [Zn(NO_3_)L^1^Dy(NO_3_)_2_(H_2_O)] shows more than eight expected transitions, resulting from the splitting of ^6^H_15/2_ ground state into eight Kramers doublets (*J* + 1/2). This indicates the presence of “hot bands” arising from the first excited state of the emitting level ^7^F_9/2_ (Figure 2). Deconvolution of the emission bands, using Gaussian functions, allows one to experimentally obtain the crystal-field splitting of the Kramers doublets. Hence, the gap between the ground and first excited doublets is estimated at 48 cm^−1^, in line with the value of Δ = 35 cm^−1^ obtained by alternate currents (ac) magnetic measurements. This suggests that the relaxation occurs *via* the first excited Kramers doublet corresponding to an Orbach process. Nevertheless, the slight discrepancy between luminescence and magnetism indicates that additional magnetic relaxation mechanisms are involved. This is further corroborated through the study of both the magnetic and luminescence properties of the aforementioned complex, diluted in a diamagnetic yttrium matrix (Long et al., 2016b). Photoluminescence confirms that: (i) the Dy^3+^ ion remains in a similar environment upon chemical dilution; (ii) as expected, the energy gap between the ground and first excited doublets is identical. The magnetic measurements for the diluted sample reveal an increased anisotropic barrier of Δ = 45 cm^−1^ due to the removal of the dipolar interactions, known to enhance the QTM, that decrease the effective barrier. The value of Δ is in remarkable accordance with that obtained by luminescence, which confirms that such an approach can be used to compare the results from these two experimental techniques and further shed light on the mechanisms that govern the slow relaxation of magnetization.

Such simple methodology has been widely extended, by other groups and including our own, to numerous SMM or coordination networks that exhibit a single-ion based magnetic relaxation in order to obtain a detailed picture of the lanthanide crystal-field and therefore improves comprehension of the relaxation dynamics (Long et al., 2018b; Jia et al., 2019). We would also like to emphasize that such outcomes could also be further confirmed by the decisive input from *ab initio* calculations, especially with systems that exhibit multiple crystallographically independent sites (Long et al., 2015).

Pure 4*f* Schiff base complexes also represent another class of promising systems. However, it remains difficult to simultaneously increase the magnetic anisotropy to generate a genuine slow relaxation of magnetization, while retaining the lanthanide luminescence (Shintoyo et al., 2014; Long et al., 2018a). Therefore, one alternative strategy consists of synthesizing heterotrinuclear Zn_2_Dy complexes (Oyarzabal et al., 2015; Sun et al., 2016) in which the lanthanide ion is sandwiched between two bis-phenoxide moieties. This results in an enhancement of the axial crystal-field. The five-membered ring constituted by four methoxy oxygen and one solvate, or counter-ion, defines a basal plane (hard plane) almost perpendicular to the Zn^2+^-Dy^3+^-Zn^2+^ arrangement. Numerous complexes with various counter-anions and co-ligands that exhibit SMM behavior and Dy^3+^-based luminescence associated with remanent emission from the ligands, have been reported (Costes et al., 2015, 2016). To this point, we have recently described an example of a luminescent trinuclear complex [(ZnL^1^Cl)_2_Dy(H_2_O)]_4_[ZnCl_4_]_2_·H_2_O that shows a zero-field slow relaxation of magnetization that could be observed for up to 30 K (Boulkedid et al., 2018). The compound exhibits the typical Dy^3+^ emission, but the presence of four different crystallographically independent dysprosium sites precludes the extraction of the energy difference between the ground and first excited doublets. Luminescence reveals however a large total crystal-field splitting of about 1,500 cm^−1^, indicating that such systems may have great potential if the relaxation occurs through higher excited Kramers doublets.

Schiff-base ligands could also be employed to introduce further functionalities. For instance, implementing chirality, opens the field to new properties that result from the crystallization in non-centrosymmetric structures. One can cite for instance Natural Circular Dichroism (NCD), Second-Harmonic Generation (SHG), Circular Polarized Luminescence (CPL) as well as magneto-optical cross-effects that result from the interplay with magnetism, such as magneto-chiral dichroism and Magnetized Second-Harmonic Generation (MSHG) (Train et al., 2011). In addition, the crystallization in appropriate non-centrosymmetric space groups paves the way toward advanced electrical properties such as piezo/pyroelectricity and ferroelectricity. With this in mind, we reported chiral [ZnL^2^Dy(OAc)(NO_3_)_2_] complexes based on the enantiopure Schiff base ligands *R*,*R* or *S*,*S*-HL_2_ = phenol,2,2′[2,2-diphenyl-1,2-ethanediyl]bis[(E)-nitrilomethylidyne]-bis(6-methoxy) (Long et al., 2015). Each enantiomer crystallizes in the polar *P*2_1_ space group, with two crystallographically inequivalent homochiral Zn^2+^/Dy^3+^ complexes, in the asymmetric unit. Apart from the typical dysprosium luminescence and slow relaxation of the magnetization that have been correlated and compared with results obtained from *ab initio* calculations, the compounds exhibit a ferroelectric behavior up to the decomposition of the material at 300°C, making it the highest temperature at which a switchable polarization has been observed for a molecular ferroelectric. Such robust chiral molecular compounds may represent alternative candidates for high-temperature ferroelectrics (Hang et al., 2011).

## Conclusions and Future Outlook

Schiff-bases represent an interesting class of ligands from which to design luminescent lanthanide SMM. Their infinite diversity and flexibility make them ideal candidates to face the challenges in the field and to obtain air-stable luminescent SMM with high energy barriers. In a more general context, the in-depth understanding of magnetic relaxation in lanthanide-based SMM remains a challenge as this involves concepts and models in physics from the 60's, related for instance to spin-phonon coupling (Escalera-Moreno et al., 2018), which should be modernized. In this regard, photoluminescence could definitely shed light on lanthanide crystal-field splitting, to determine if the relaxation proceeds *via* the 1st or higher excited states, or involves underbarrier Raman, direct or QTM processes. Moreover, studying the vibronic coupling in high temperature SMM may be experimentally achieved by photoluminescence (emission lifetimes, *f*-*f* relative intensities…).

While the correlation between magnetism and photoluminescence should be viewed as the inception, studying the interplay between the two properties constitutes a major milestone. As both properties are intimately correlated to the electronic structure of the lanthanide ion, the coupling is expected to be strong and such a cross-effect has previously been evidenced more than 50 years ago, in paramagnetic ytterbium garnet (Buchanan et al., 1967; Grünberg et al., 1969). Thus, applying a magnetic field induces a strong modification of the emission spectrum and has been explained by the well-known Zeeman effect, that lifts the degeneracy of the Kramers doublets. Such approach was first demonstrated in lanthanide SMM, in 2016 (Bi et al., 2016) in which values of the gyromagnetic factor (that are usually difficult to experimentally obtain) were extracted, confirming the gap between the first and excited Kramers doublets. One point that needs to be addressed concerns the comprehension of the field dependence of the emission intensity as observed in others SMM (Chen et al., 2017). Such breakthroughs confirm the possibility to control both the emission intensity and wavelength by a magnetic field, which could be relevant for applications such as magnetic field sensors. However, such inductive effects may simply occur in any luminescent 4*f* paramagnetic compound, therefore, future studies should examine the existence of an interplay between the SMM property (magnetic bistability) and the luminescence. This requires circumventing synthetic issues to design systems, simultaneously exhibiting a significant magnetic remanence and coercivity at a pertinent temperature with stability over a long-time scale. This last point is of critical importance since 4*f* SMM usually shows quick relaxation of magnetization, due to the QTM. Major advances have recently been achieved in organometallic SMM, showing magnetic bistability of up to 60–80 K (Goodwin et al., 2017; Guo et al., 2017, 2018), confirming that the design of high performing luminescent SMM with possible air-stability is within reach if suitable sensitizer ligands that are able to maximize the magnetic anisotropy, are rationally conceived.

On the other hand, introduction of chirality and other properties, resulting from the non-centrosymmetric character of the crystal structures, may be easily achieved using Schiff base ligands. Simple multifunctional molecular ferroelectrics therefore represent ideal candidates to study the coupling between the constitutive functionalities such as the magneto-electrical coupling. Designing a strong coupling between an electric and magnetic property clearly constitutes an important challenge in the field of solid-state chemistry (Fiebig et al., 2016). This indicates that molecular systems may control polarization, by applying a magnetic field and *vice versa* with prospective applications in non-volatile memories and low-consumption devices (Eerenstein et al., 2006; Cheong and Mostovoy, 2007; Mandal et al., 2015).

More generally and in order to fulfill these ambitious objectives, molecular materials also need to be integrated or shaped into more complex architectures (surfaces, films, or composites) for use in practical applications. Such strategies necessitate investigating that the considered molecular objects and their associated functionalities remain preserved. Among the characterization techniques used to investigate the latter, photo-luminescence could easily be used for this purpose. Close collaboration between chemists and photo physicists is therefore clearly necessary, in order to achieve these objectives.

## Author Contributions

The author confirms being the sole contributor of this work and has approved it for publication.

### Conflict of Interest Statement

The author declares that the research was conducted in the absence of any commercial or financial relationships that could be construed as a potential conflict of interest.
